# P-929. Identification and Treatment Outcomes of Patients with Infective Endocarditis Coinfected with Hepatitis C Virus

**DOI:** 10.1093/ofid/ofae631.1120

**Published:** 2025-01-29

**Authors:** Lauren C McQuaide, Sami El-Dalati, Bobbi Jo Walston

**Affiliations:** University of Kentucky College of Pharmacy, Lexington, Kentucky; University of Kentucky, Lexington, Kentucky; UNC Medical Center, Durham, NC

## Abstract

**Background:**

Patients with infective endocarditis (IE) often have co-morbid injection substance use and are at risk for infection with hepatitis C virus (HCV). As of 2023, only 10% of patients who inject drugs have been treated for HCV in the United States. Low rates of treatment may reflect the logistical challenges associated with HCV therapy. To improve access to treatment for patients with IE and HCV, we developed an interdisciplinary collaboration between pharmacy and clinical infectious diseases (ID) providers.

**Figure:**
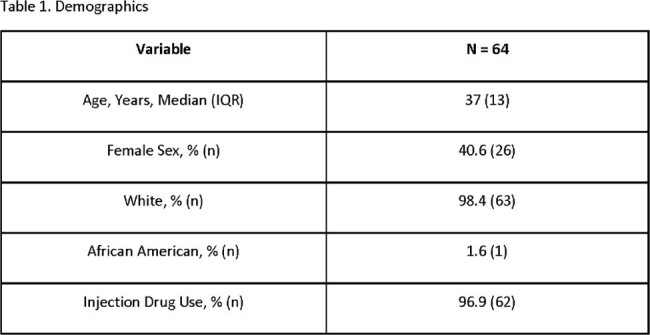

**Methods:**

The University of Kentucky (UK) implemented a cardiovascular ID consult service in September 2021. The service including physicians, advanced practice providers, a pharmacist and nurse. The consult team worked with UK Healthcare specialty pharmacy to create a pathway connecting patients with IE and HCV to treatment and outpatient follow-up. Regimens included either glecaprevir/pibrentasvir for 8 weeks or sofosbuvir/velpatasvir for 12 weeks. IRB approval was obtained from UK. Study investigators then retrospectively reviewed the endocarditis team registry to identify patients referred for HCV treatment and collect demographic and outcomes related data.

**Figure:**
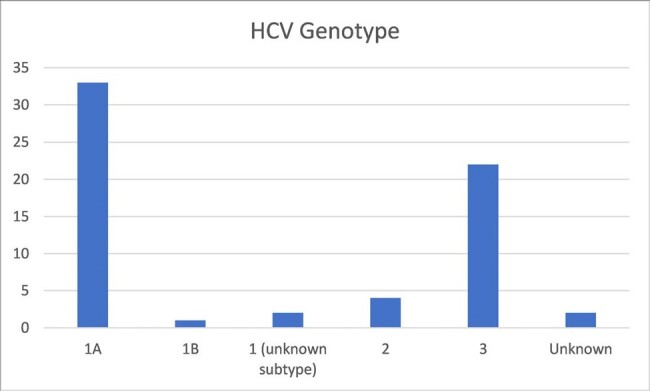

**Results:**

Between September 2021 and April 2024, 64 patients with IE and HCV who were referred for expedited treatment were identified. The mean age was 37.9 years and 40.6% were female. Sixty-two patients had recent injection drug use. Only 1 was treatment experienced and 51.6% had HCV genotype 1A. Seventy-five percent (48) started treatment and 57.8% were prescribed sofosbuvir/velpatasvir. Thirty-three of 48 (68.8%) patients who started treatment completed therapy. Of the 15 patients who did not complete treatment,10 were lost to follow-up and 3 had adverse drug reactions. Twenty-two of 48 had viral loads collected for SVR12 and 20 were negative.

**Figure:**
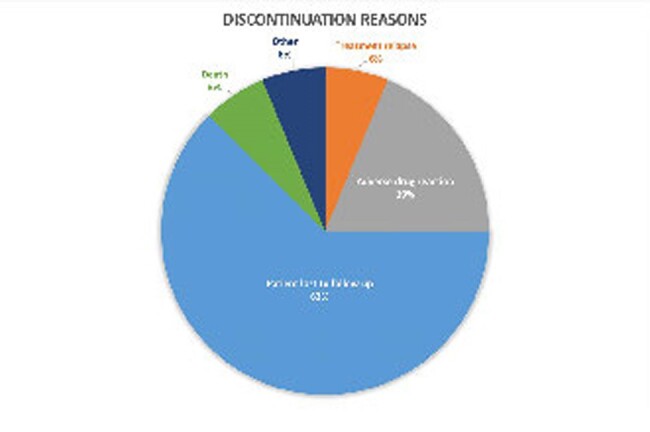

**Conclusion:**

Using a standardized, interdisciplinary approach, 31.3% of patients with IE and HCV completed treatment with documented evidence of SVR12. An additional 17% of patients completed therapy but were unable to complete testing for SVR12. Loss to follow-up remains the primary barrier to starting therapy and accurately recording treatment outcomes. Future efforts should focus on ease of access to treatment and follow-up.

**Disclosures:**

**All Authors**: No reported disclosures

